# Brain Metastases in Elderly Patients—The Role of Surgery in the Context of Systemic Treatment

**DOI:** 10.3390/brainsci11010123

**Published:** 2021-01-18

**Authors:** Martin Proescholdt, Stephanie T. Jünger, Petra Schödel, Karl-Michael Schebesch, Christian Doenitz, Tobias Pukrop, Julius Höhne, Nils-Ole Schmidt, Martin Kocher, Holger Schulz, Maximilian Ruge, Kevin König, Roland Goldbrunner, Stefan Grau

**Affiliations:** 1 Department of Neurosurgery, University Hospital Regensburg, Franz Josef Strauß Allee 11, 93053 Regensburg, Germany; Petra.Schoedel@ukr.de (P.S.); Karl-Michael.Schebesch@ukr.de (K.-M.S.); christian.doenitz@ukr.de (C.D.); Julius.Hoehne@ukr.de (J.H.); Nils-Ole.Schmidt@ukr.de (N.-O.S.); 2 Wilhelm Sander Neuro-Oncology Unit, University Hospital Regensburg, Franz Josef Strauß Allee 11, 93053 Regensburg, Germany; tobias.pukrop@ukr.de; 3 Department of General Neurosurgery, University Hospital Cologne, Kerpener Straße 62, 50937 Cologne, Germany; stephanie.juenger@uk-koeln.de (S.T.J.); Kevin.koenig@uk-koeln.de (K.K.); roland.goldbrunner@uk-koeln.de (R.G.); stefan.grau@uk-koeln.de (S.G.); 4 Department of Oncology, University Hospital Regensburg, Franz Josef Strauß Allee 11, 93053 Regensburg, Germany; 5 Department of Stereotaxy and Functional Neurosurgery, University Hospital Cologne, Kerpener Straße 62, 50937 Cologne, Germany; martin.kocher@uk-koeln.de (M.K.); maximilian.ruge@uk-koeln.de (M.R.); 6 PIOH, Clinic for Oncology and Hematology, Kölner Straße 9, 50226 Frechen, Germany; hschulz@pioh.de

**Keywords:** Brain metastases, elderly patients, targeted therapy, survival

## Abstract

In patients with brain metastases (BM), advanced age is considered a negative prognostic factor. To address the potential reasons for that, we assessed 807 patients who had undergone BM resection; 315 patients aged at least 65 years (group A) were compared with 492 younger patients (group B). We analyzed the impact of the pre- and postoperative Karnofsky performance status (KPS), postoperative treatment structure and post-treatment survival. BM resection significantly improved KPS scores in both groups (*p* = 0.0001). Median survival after BM resection differed significantly between the groups (A: 5.81 *vs*. B: 8.12 months; *p* = 0.0015). In both groups, patients who received postoperative systemic treatment showed significantly longer overall survival (*p* = 0.00001). However, elderly patients less frequently received systemic treatment (*p* = 0.0001) and the subgroup of elderly patients receiving such therapies had a significantly higher postsurgical KPS score (*p* = 0.0007). In all patients receiving systemic treatment, age was no longer a negative prognostic factor. Resection of BM improves the functional status of elderly patients, thus enhancing the likeliness to receive systemic treatment, which, in turn, leads to longer overall survival. In the context of such a treatment structure, age alone is no longer a prognostic factor for survival.

## 1. Introduction

Due to the demographic change, the proportion of elderly people in the total population is growing significantly [1]. Since the incidence of cancer increases with age [2] and brain metastases (BM) more often occur in older patients with cancer [3], health care providers are faced with a growing number of elderly patients with BM [4]. Because advanced age and impaired independency are associated with poor outcome in patients with BM, age and functional status have become integral parameters of established classification systems for selecting patients for surgical and adjuvant treatment [5,6,7]. However, several reports have shown that surgical resection of BM significantly improves pre-surgical Karnofsky performance status (KPS) scores [8,9,10,11,12,13] and this improvement may also apply to elderly patients with BM. Furthermore, the introduction of novel molecular treatment modalities such as targeted therapies and immune-checkpoint inhibitors [14] have rapidly changed treatment concepts for elderly patients with BM. Thus, patients who would have been previously classified as patients with poor prognosis because of the presence of multiple metastases or advanced age [15,16,17] may also benefit from these advances in local and systemic therapy. From a neurosurgical point of view, advanced age is associated with higher complication rates after surgical interventions because of age-associated co-morbidities [18,19,20]. Thus, the risk-benefit ratio for elderly patients in the context of surgical treatment indication is frequently debated. The aim of our study was to compare the impact of BM resection surgery between a cohort of elderly patients (>65 years) and a cohort of younger patients in the setting of modern interdisciplinary cancer treatment.

## 2. Results

### 2.1. Demographics

Between 2012 and 2018, 807 consecutive patients had undergone surgery for BM; 315 patients were at least 65 years of age (group A) and 492 patients were younger (group B). Baseline characteristics of the entire study population and differences between the two age groups are reported in Table 1. The most frequent primary tumor was lung cancer (41.5%) followed by breast cancer (15.4%) and malignant melanoma (14.6%). The distribution of the primary tumor entities differed significantly between the two groups; group A showed a higher number of BM due to lung and gastrointestinal cancer and group B a higher number of BM due to breast cancer and malignant melanoma (*p* = 0.001; Table 1). The higher proportion of BM due to breast cancer in the group of younger patients indicates a significantly higher number of female patients in group B; hence, the two groups also differed with regard to their sex ratio. Group A had a higher proportion of patients with controlled systemic disease (*p* = 0.042) and solitary BM (*p* = 0.0001) than group B. Correspondingly, the number of BM per patient was lower in group A (*p* = 0.002). As expected, the Charlson comorbidity score (CCS) was higher in group A than in group B (4.0 *vs.* 3.0; *p* = 0.0001); however, neither surgical morbidity (*p* = 0.172) nor mortality (*p* = 0.534) was associated with a higher CCS in the group of elderly patients. It should provide a concise and precise description of the experimental results, their interpretation as well as the experimental conclusions that can be drawn.

### 2.2. Surgical Outcome and Complications

In the case of multiple BM, 64 (65.3%) patients in group A underwent resection of 1 lesion and 34 (34.7%) patients resection of 2 lesions; the corresponding figures in group B were 119 (56.6%) patients (1 lesion) and 91 (43.4%) patients (2 lesions) (*p* = 0.189). Complete BM resection was documented by means of early postoperative MRI in 274 (86.9%) patients in group A and 424 (86.2%) patients in group B (*p* = 0.774). Median preoperative KPS was similar in the two groups (both groups median KPS score of 80, range: 40–100; *p* = 0.128; Table 1). Resection of BM postoperatively increased median KPS scores of the entire population to 90 (40–100) (*p* = 0.0001), an improvement that was achieved in group A (*p* = 0.0001) as well as in group B (*p* = 0.0001). In patients with a presurgical KPS score of less than 100, postsurgical KPS scores were improved in 49.5% of the older patients and in 54.7% of the younger patients. Consequently, 93 (11.5%) of all patients showed improved recursive partitioning analysis (RPA) allocation after surgery, an improvement rate that did not differ between the two groups (A = 11.1% *vs.* B = 12.0%; *p* = 0.769). In group A, pre- and postoperative allocation to RPA groups shifted from class III to class II in 35 patients (11.1%) and in group B from class III to class I in 22 patients (4.5%); from class III to class II in 33 patients (6.7%) and from class II to class I in 4 patients (0.8%). The presurgical Medical Research Council-Neurological Performance Status Scale (MRC - NPS) was not different between the age groups (*p* = 0.227) and was improved post surgically in 69.8% and 70.0% and in group A and B respectively (*p* = 0.965). Surgical complications occurred in 87 (10.8%) patients (group A: *n* = 34; 10.9%, group B: *n* = 53; 10.7%, *p* = 0.992). 34 patients died during the first 30 days after surgery, accounting for a peri-operative mortality rate of 4.2% (group A: *n* = 20; 6.4%, group B: *n* = 14; 2.8%, *p* = 0.016). 50 patients (6.2%) of the entire population (group A: *n* = 13; 4.1%, group B: *n* = 37; 7.5%, *p* = 0.051) developed minor complications such as wound healing disorders (*n* = 44 patients) or cerebro-spinal fluid fistulas (CSF) (*n* = 7 patients) and 1 patient developed both wound healing and a CSF.

### 2.3. Postsurgical Treatment Patterns

**Figure 1:**
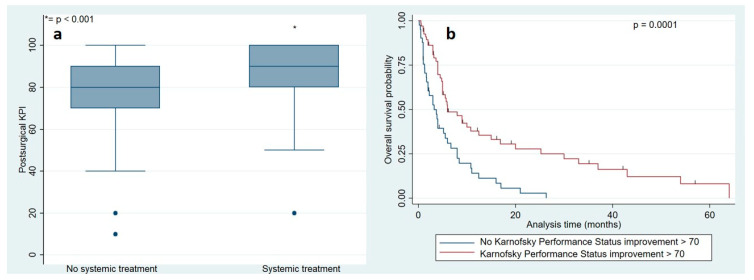
(**a**) The postsurgical KPS was significantly better in patients receiving systemic treatment, illustrating the impact of functional status on the decision whether to apply systemic treatment or not. (**b**) Elderly patients with a KPS score of less than 70 who experienced functional recovery after BM resection above a KPS score of 70 showed significantly better overall survival (median OS: 10.73 *vs.* 5.22 months, *p* = 0.0001).

**Figure 2:**
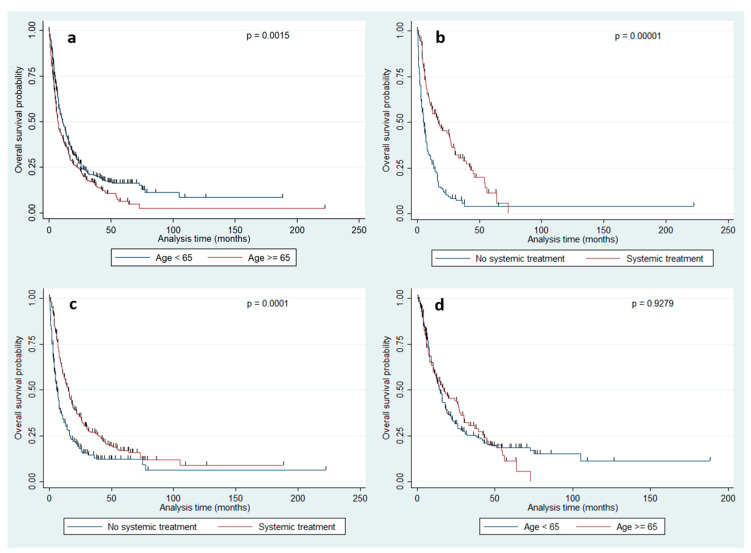
(**a**) Overall survival of the entire population (*n* = 807) stratified by age group (younger Table 65. years *vs.* 65 years and older). The elderly population shows a significantly poorer overall survival (median OS: 5.81 *vs.* 8.83; *p* = 0.0015). (**b**) The comparison of overall survival between patients who received systemic treatment or not applied to the entire population showed a significantly better outcome in patients receiving such treatment (median OS: 15.38 *vs.* 6.99 months, *p* = 0.00001). (**c**) The identical comparison applied to the elderly population confirmed the effects of systemic effect on overall survival (median OS: 10.33 *vs.* 3.87 months, *p* = 0.0001). (**d**) When analyzing only patients who received systemic treatment, no significant impact of age group was detectable anymore (*p* = 0.927).

124 (15.4%) patients did not receive adjuvant local radiotherapy and the number of these patients was significantly higher in group A (*n =* 63; 20.0%) than in group B (*n* = 61; 12.4%; *p* = 0.009). Similarly, a significantly larger proportion of older patients than younger patients did not receive adjuvant systemic treatment (*n* = 173, 54.9% *vs. n* = 197, 40.1%; *p* = 0.0001). In patients with impaired MRC-NPS (*n* = 479, 59.4%), improvement of neurological function was significantly associated with a higher rate of adjuvant treatment both in group A (76.5% *vs.* 23.5%, *p* = 0.003) and B (75.7% *vs.* 24.3%; *p* = 0.006). In both groups, median postsurgical KPS scores were significantly higher in patients who received adjuvant radiation or systemic treatment or both (90 *vs.* 80; *p* = 0.0001 in all group comparisons; for group A and systemic treatment: Figure 1a). This finding indicates that the postsurgical KPS score is a relevant factor for selecting patients for adjuvant treatment. Therefore, patients with a post surgically improved KPS score had a significantly higher chance of receiving adjuvant systemic treatment than patients without an improved KPS score (60.2% *vs.* 39.8%, *p* = 0.004). This effect was similar between the two age groups (group A: 59.8% *vs.* 40.2%; *p* = 0.029 *vs.* group B: 60.3% *vs.* 39.7% *p* = 0.032). Patients in group A who had a post surgically improved KPS score lived significantly longer than patients without an improved KPS score (*p* = 0.0001, Figure 1b). Multiple logistic regression using postsurgical systemic treatment as an outcome variable showed age and postsurgical (but not presurgical) KPS scores to be independent factors for the decision on adjuvant systemic therapy (*p* = 0.0001). In addition, patients of both groups who received postoperative systemic treatment showed significantly longer overall survival (4.73 *vs.* 11.81 months, *p* = 0.00001, Figure 2b) than patients without such treatment. Interestingly, when only analyzing patients who received systemic treatment, we no longer detected any difference in overall survival between the two age groups (*p* = 0.927, Figure 2d).

### 2.4. Survival Outcome

At the time of analysis, 573 (71.0%) patients had died. Median overall survival time was 7.12 months. According to univariate analysis of the entire population, significant parameters for overall survival were pre- and postsurgical KPS scores, age and age group (A *vs*. B, Figure 2a), adjuvant radiation and systemic treatment (Figure 2b), tumor site, metastasis status (solitary and singular *vs*. multiple), the timing of BM (synchronous *vs*. metachronous) and control of the systemic disease (Table 2). The multivariate cox regression model showed pre- and postsurgical KPS scores, age group, metastasis status and tumor site to be independent prognostic factors for survival in the entire population (Table 3). Stratified by age group, adjuvant radiation and systemic treatment (Figure 2c), tumor site, the interval between tumor diagnosis and detection of BM, metastasis timing (synchronous *vs.* metachronous) and pre- and postsurgical KPS scores were associated with overall survival in the univariate analysis in group A (Table 2). All above-mentioned parameters except for tumor site and metastasis timing remained significant in the multivariate analysis (Table 4). In contrast, univariate analysis of group B showed disease control and metastasis status as significant factors in addition to the factors found to be significant in the group of older patients. However, tumor site, the interval between tumor diagnosis and detection of BM and the timing of metastasis—which were significant univariate factors in elderly patients—were not associated with survival in younger patients (Table 2). Multivariate analysis showed postsurgical KPS scores and—in contrast to the group of older patients—disease control and metastasis status as independent prognostic factors (Table 5).

## 3. Discussion

Several studies have shown that advanced age is a prognostic factor for poor overall survival in patients undergoing surgical BM resection [18,21,22,23,24,25], which was corroborated by the findings of the current study. However, when looking at the postsurgical treatment pattern, we found that a large proportion of elderly patients did not receive any adjuvant systemic treatment, especially patients with a poor postsurgical KPS score. Such treatment is more likely to be given to patients after the improvement of their functional status by means of surgical BM resection, which, in turn, significantly improves overall survival. In fact, when analyzing all patients who received adjuvant systemic treatment, the age-related difference in overall survival was no longer detectable. The positive impact of BM resection on the functional status has been illustrated in several reports [8,9,10,11,12,26] but our study is the first to show that KPS scores improved by BM resection is associated with intensified postsurgical treatment and survival outcome in elderly patients. The general assumption is that advanced age is associated with a higher incidence of treatment-related toxicity, which leads to poorer outcome [27]. However, several studies have indicated that elderly patients with cancer may also benefit from intensified treatment and that they may simply be undertreated due to clinical decisions based on chronological age [28,29]. Notably, toxicity and efficacy data of modern targeted treatments in elderly patients are scarce, since this age group is underrepresented in clinical cancer trials [30,31,32], making patient selection for such treatments even more difficult. A recent study describing the treatment pattern of elderly patients with breast cancer-derived BM has shown a general increase in adjuvant treatment rates over the past 20 years. However, only 18% of patients received combined treatment consisting of resection, radiation or systemic treatment or both, in the most recent treatment period [15]. Another argument against intensified treatment strategies in elderly patients is the physiological change associated with advanced age and the resulting comorbidities [33]. Although we found a higher Charlson comorbidity score in the group of elderly patients, we did not observe any correlation between comorbidities and survival. In addition, a higher comorbidity status was not associated with the surgical complication rate in our elderly patients. As a confirmation of our results, a surgical trial analyzing the safety of awake craniotomy in elderly patients failed to show an increased rate of surgical morbidity [34]. However, in contrast to the above-mentioned study, we did observe a significantly higher surgical mortality rate in elderly patients. Of all elderly patients who had died in the early postoperative period, 25% showed postoperative intracranial hemorrhage (compared to an overall postoperative hemorrhage rate of only 4.1% in the group pf elderly patients). Importantly, each of these patients had received anticoagulation therapy before BM resection because of cardiovascular comorbidity. Sensitivity analyses omitting postoperative hemorrhage as a complication no longer show any significant difference in surgical mortality between age groups, thus highlighting pretreatment with anticoagulants as a specific risk factor that requires special attention in the clinical management of elderly patients with BM. The retrospective design is a potential limitation of our study since we cannot entirely rule out a clinical selection bias. However, when carefully considering comorbidities and the consecutive surgical risk profile, elderly patients do functionally benefit from surgical resection which leads to a higher likeliness to receive adjuvant treatment and possibly better outcome.

## 4. Patients and Methods

We retrospectively analyzed 807 consecutive patients of two large University Medical Centers (Cologne and Regensburg), who had undergone neurosurgical resection of BM between 2012 and 2018. The study was approved by the respective local Ethics Committees (Cologne approval no. 18-089, Regensburg approval no.19-1546-101). Baseline clinical and therapy-related parameters of the patients were obtained from electronic and paper-based patient charts. The Charlson comorbidity score (CCS) was calculated [35]. BM resection had been decided by institutional interdisciplinary tumor boards. Metastatic tissue was removed by means of micro-neurosurgical techniques including neuro-navigation, fluorescence support, intraoperative ultrasound guidance and, if required, intraoperative electrophysiological monitoring. The extent of resection was determined by postoperative contrast-enhanced cranial magnet resonance imaging (MRI) carried out within 24 to 48 hours after surgery. Follow-up data were extracted from electronic patient charts of the institutional outpatient clinic and paper-based communication from the treating oncologists. Patients were excluded from analysis if they had previously been treated for BM or in the case of missing data on oncological treatment after BM resection. Statistical calculations were done using Stata 14 (StataCorp, College Station, TX, USA). For descriptive statistics, continuous values are given as mean, median and range and ordinal and categorical variables are stated as counts and percentages. Survival rates were estimated using the Kaplan-Meier method. Univariate analysis (Log-rank test) was used to identify covariates with an impact on overall survival after BM resection. The following parameters were analyzed: primary tumor type, status of BM (singular or solitary *vs*. multiple), timing (synchronous *vs*. metachronous), pre- and postoperative KPS scores, pre- and postoperative MRC-NPY [13], adjuvant radiation treatment and postoperative systemic treatment (molecular therapy including immunotherapy, targeted therapy and chemotherapy). Multivariate Cox hazards regression analysis was used for factors that were significant in the univariate analysis. *P*-values below 0.05 were considered statistically significant.

## 5. Conclusions

In conclusion, we could show that surgical resection of BM improves the functional status in a large proportion of elderly patients, which leads to a higher chance of receiving adjuvant treatment and to longer overall survival. Therefore, the traditional paradigm of age being a negative prognostic factor per se must be questioned in the context of a modern, multidisciplinary treatment structure.

## Figures and Tables

**Table 1:** Baseline characteristics of the entire patient population and stratified into age groups.

Characteristics	Total	Age Group		*p*-Value
		>= 65 (A)	< 65 (B)	
*N*	807	315	492	
Age (years) Mean/median Range	60.6/61.0 29.8–85.9	71.2/72.1 65–85.9	53.6/55.8 25.3–64.9	
Gender Female (%) Male (%)	51.05 48.95	45.1 54.9	54.9 45.1	0.0007
Primary cancer sites (%) Lung Breast Melanoma GI tract Kidney Others	41.5 15.4 14.6 9.2 3.7 15.6	44.4 12.4 11.4 11.4 5.1 15.3	39.6 17.9 16.7 7.7 2.9 15.2	0.001
Presurgical Karnofsky performance status Median Range	80 40–100	80 40–100	80 40–100	0.128
Postsurgical Karnofsky performance status Median Range	90 50–100	90 50–100	90 60–100	0.0002
Systemic disease control Yes (%) No (%)	42.8 57.2	49.3 50.7	38.5 61.5	0.042
Metastasis status (%) Solitary Singular Multiple	22.9 38.9 38.2	30.5 38.4 31.1	18.1 39.2 42.7	0.0001
Number of metastases Mean/median Range	2.1/1 1–19	1.8/1 1–19	2.3/1 1–17	0.002
Metastasis timing (%) Synchronous Metachronous	36.4 63.6	37.5 62.5	35.8 64.2	0.327
Comorbidity score Mean/median Range	3.5/3.0 0–9	4.3/4.0 1–9	2.9/3.0 0–9	0.0001
Interval between tumor diagnosis to BM Mean/median Range	30.4/11.1 0–139	28.3/10.0 0–132	31.74/12.9 0–139	0.392
Postsurgical radiation (%)	86.6	80.0	87.6	0.009
Postsurgical systemic treatment (%)	49.2	45.1	59.9	0.0001
Surgical morbidity (%)	10.8	10.9	10.7	0.992
Surgical mortality	4.2	6.4	2.9	0.016

**Table 2:** Univariate analysis of factors associated with overall survival.

	Entire Population	Age Group	
		>= 65 (A)	< 65 (B)
Parameter	*p*-Value	*p*-Value	*p*-Value
Group A *vs*. B	0.0015		
Age	0.008	0.067	0.555
Tumor location	0.002	0.0005	0.161
Gender	0.071	0.159	0.691
Comorbidity score	0.604	0.859	0.123
Primary tumor	0.480	0.809	0.062
Disease control	0.013	0.129	0.011
Metastasis timing	0.021	0.035	0.226
Metastasis status	0.009	0.251	0.005
Number of metastases	0.152	0.321	0.173
Interval between tumor diagnosis to BM	0.225	0.030	0.962
Postsurgical radiation	0.0001	0.0001	0.0001
Postsurgical systemic treatment	0.0001	0.0001	0.0001
Presurgical Karnofsky performance status	0.0001	0.012	0.0001
Postsurgical Karnofsky performance status	0.0001	0.001	0.0001
Extent of resection (complete *vs*. incomplete)	0.257	0.219	0.496

**Table 3:** Cox proportional hazards regression analysis shows age group, tumor location, metastasis status, pre- and postsurgical Karnofsky performance status (KPS) as independent prognostic factors for overall survival in the entire population.

Parameter	Hazard Ratio	95% CI		*p*-Value
Presurgical Karnofsky performance status	0.982	0.972	0.992	0.001
Metastasis status	1.264	1.076	1.486	0.004
Group A/B	1.369	1.070	1.751	0.012
Tumor location	1.066	1.014	1.121	0.012
Postsurgical Karnofsky performance status	0.990	0.981	1.000	0.038
Metastasis timing	0.784	0.604	1.017	0.067
Postsurgical radiation	0.820	0.662	1.017	0.072
Postsurgical systemic treatment	0.845	0.700	1.020	0.080
Disease control	0.917	0.702	1.199	0.530

**Table 4:** Cox proportional hazard regression analysis shows pre and even more pronounced the postsurgical KPS, interval between tumor diagnosis and brain metastases (BM) detection, adjuvant radiation and systemic treatment as independent prognostic factors for overall survival in the elderly population.

Parameter	Hazard Ratio	95% CI		*p*-Value
Interval tumor diagnosis BM	0.996	0.993	0.999	0.040
Metastasis timing	0.811	0.632	1.041	0.101
Presurgical Karnofsky performance status	0.989	0.980	0.999	0.041
Postsurgical Karnofsky performance status	0.983	0.975	0.992	0.0001
Postsurgical radiation	0.753	0.580	0.978	0.034
Postsurgical systemic treatment	0.636	0.509	0.795	0.0001
Tumor location	1.041	0.985	1.110	0.133

**Table 5:** Cox proportional hazard regression analysis shows presurgical KPS, metastasis status and control of systemic disease as independent prognostic factors for overall survival in the population younger than 65 years.

Parameter	Hazard Ratio	95% CI		*p*-Value
Disease control	0.653	0.469	0.910	0.012
Metastasis status	1.260	1.087	1.460	0.002
Presurgical Karnofsky performance status	0.992	0.983	1.001	0.102
Postsurgical Karnofsky performance status	0.984	0.973	0.996	0.010
Postsurgical radiation	0.707	0.549	0.910	0.007
Postsurgical systemic treatment	0.751	0.613	0.920	0.006

## Data Availability

The data presented in this study are available on request from the corresponding author. The data are not publicly available due to ethical and statutory data protection rules.
